# Generalized Concentration Addition Predicts Joint Effects of Aryl Hydrocarbon Receptor Agonists with Partial Agonists and Competitive Antagonists

**DOI:** 10.1289/ehp.0901312

**Published:** 2009-12-22

**Authors:** Gregory J. Howard, Jennifer J. Schlezinger, Mark E. Hahn, Thomas F. Webster

**Affiliations:** 1 Environmental Health Department, Boston University School of Public Health, Boston, Massachusetts, USA; 2 Environmental Studies Department, Dickinson College, Carlisle, Pennsylvania, USA; 3 Biology Department, Woods Hole Oceanographic Institution, Woods Hole, Massachusetts, USA

**Keywords:** additivity, AhR, aryl hydrocarbon receptor, concentration addition, interaction, mixtures, TEF

## Abstract

**Background:**

Predicting the expected outcome of a combination exposure is critical to risk assessment. The toxic equivalency factor (TEF) approach used for analyzing joint effects of dioxin-like chemicals is a special case of the method of concentration addition. However, the TEF method assumes that individual agents are full aryl hydrocarbon receptor (AhR) agonists with parallel dose–response curves, whereas many mixtures include partial agonists.

**Objectives:**

We assessed the ability of generalized concentration addition (GCA) to predict effects of combinations of full AhR agonists with partial agonists or competitive antagonists.

**Methods:**

We measured activation of AhR-dependent gene expression in H1G1.1c3 cells after application of binary combinations of AhR ligands. A full agonist (2,3,7,8-tetrachlorodibenzo-*p*-dioxin or 2,3,7,8-tetrachlorodibenzofuran) was combined with either a full agonist (3,3′,4,4′,5-pentachlorobiphenyl), a partial agonist (2,3,3′,4,4′-pentachlorobiphenyl or galangin), or an antagonist (3,3′-diindolylmethane). Combination effects were modeled by the TEF and GCA approaches, and goodness of fit of the modeled response surface to the experimental data was assessed using a nonparametric statistical test.

**Results:**

The GCA and TEF models fit the experimental data equally well for a mixture of two full agonists. In all other cases, GCA fit the experimental data significantly better than the TEF model.

**Conclusions:**

The TEF model overpredicts effects of AhR ligands at the highest concentration combinations. At lower concentrations, the difference between GCA and TEF approaches depends on the efficacy of the partial agonist. GCA represents a more accurate definition of additivity for mixtures that include partial agonist or competitive antagonist ligands.

Dioxin-like compounds [e.g., non-*ortho*- substituted polychlorinated biphenyls (PCBs), chlorinated dibenzo-*p*-dioxins, and chlorinated dibenzofurans] rank among the highest-priority environmental toxicants at Superfund sites (Agency for Toxic Substances and Disease Registry 2007). Because these compounds occur most commonly as complex mixtures, methods to predict the expected outcome of combination exposures are critical both to risk assessment and to an accurate judgment of whether mixture effects are additive, synergistic, or antagonistic.

A rigorous approach is to first define a model, sometimes called the null model, for the expected additive (i.e., noninteractive) effects of a combination. Mixture effects above or below those predicted can then be thought of as synergy or antagonism with respect to the null model (Kortenkamp 2007; U.S. Environmental Protection Agency 2000). Choice of the null model is crucial. An inappropriate null model for a mixture can greatly underestimate the additive (noninteractive) effects of mixtures, as was demonstrated dramatically in an experiment measuring activation of the estrogen receptor by a mixture of environmental estrogenic agents (Silva et al. 2002). Although the concentration of each individual agent was below its no observed effect concentration or effective concentration causing 1% of maximal response, the total effect of the mixture was many times greater than that predicted by a simple sum of the individual effects, a null model sometimes called effect summation. It is easy to show that the effect summation model is appropriate only for agents with linear dose–response curves (Berenbaum 1989).

Combination effects of estrogenic agents in the Silva experiment (Silva et al. 2002) were, however, accurately predicted by the null model of concentration addition (CA). CA assumes that one agent can be substituted for another in proportion to their relative potencies; it is usually thought to apply to agents that work by similar mechanisms and is not limited to linear dose–response curves (Kortenkamp 2007). CA has the added advantage of a clear graphical interpretation: Curves of constant joint effect (isoboles) must be negatively sloped straight lines when the concentration of one agent is plotted against the concentration of the other (Berenbaum 1989).

CA has an important limitation in that it cannot be applied to effect levels greater than the maximal effect achieved by the least efficacious compound included in the mixture (Silva et al. 2002). Therefore, this model cannot appropriately assess the effect of mixtures containing a partial agonist (i.e., an agonist with less than maximal efficacy). We previously derived a generalization of CA (GCA) to describe the combination effects of full agonists, partial agonists, and competitive antagonists (Howard and Webster 2009). GCA provides a definition of noninteraction that encompasses combinations with linear isoboles of any slope; for example, in the case of the Hill function dose–response curves considered here, GCA produces a distinctive pattern of linear isoboles whose slopes depend on the type of interaction being modeled (Table 1). Kinetic models of combinations of full and partial agonists acting on simple but plausible receptor systems gave results identical to the GCA model, supporting the GCA definition of additivity (Howard and Webster 2009).

The toxicity of dioxin-like agents is mediated by their interaction with the aryl hydrocarbon receptor (AhR) (Fernandez-Salguero et al. 1996). Exogenous ligands induce AhR translocation to the nucleus, dimerization with the AhR nuclear translocator protein, binding to target genes at specific DNA binding sites (AhREs), and activation of gene transcription (Denison et al. 2002). Effects of mixtures of dioxin-like compounds are estimated using the toxic equivalency factor (TEF) approach, a special case of CA (Van den Berg et al. 2006). The TEF method assumes that all individual agents are full agonists with parallel dose–response curves, differing only in potency. However, many AhR ligands, including many PCBs (Hestermann et al. 2000; Peters et al. 2006), are partial agonists.

Here, we have applied the GCA model to mixtures of AhR ligands, including full agonists, partial agonists, and antagonists. We tested the model using experimental data from binary combinations of agents, generated using a cell line stably transfected with an AhR-driven reporter construct, and comparing the results with those from the TEF model. Finally, we describe the utility of the GCA model and contrast its predictions with those of the TEF approach.

## Materials and Methods

2,3,3′,4,4′-Pentachlorobiphenyl (PCB105); 3,3′,4,4′,5-pentachlorobiphenyl (PCB126); and 2,3,7,8-tetrachlorodibenzofuran (TCDF) were purchased from Cambridge Isotope Laboratories (Andover, MA). 2,3,7,8-Tetrachlorodibenzo-*p*-dioxin (TCDD) was purchased from Ultra Scientific (North Kingstown, RI). 3,3′-Diindolylmethane (DIM) and galangin were purchased from Sigma-Aldrich (St. Louis, MO).

Purity of the PCB105 sample was assessed by gas chromatography/mass spectroscopy using a Hewlett-Packard P5989 (Hewlett-Packard, Palo Alto, CA). This analysis revealed contamination with 1.2% non-*ortho* PCB126 and 1% mono-*ortho* PCB118 (2,3′,4,4′,5-pentachlorobiphenyl).

### Measurement of AhR activation (H1G1 assay)

The H1G1.1c3 recombinant murine hepatoma cell line, kindly provided by M. Denison (University of California, Davis, Davis, California), is stably transfected with an EGFP (enhanced green fluorescent protein) reporter construct regulated by AhREs from the murine CYP1A1 promoter. H1G1.1c3 cells were cultured and prepared for experiments as described previously (Nagy et al. 2002). Briefly, H1G1.1c3 cells were plated at 2 × 10^4^ cells per well in 200 μL medium (αMEM, 10% fetal bovine serum, 50 U/mL penicillin/streptomycin) containing G418 (968 mg/L) and incubated at 37°C for 24 hr. The medium was removed and replaced with 100 μL nonselective medium before application of the test compounds.

Stock solutions of test compounds were prepared and diluted in DMSO. Each experiment used an array of seven plates, and each plate was treated with combinations of compounds. The plates were treated with vehicle (DMSO, 0.5%), a partial agonist (PCB105 or galangin), an antagonist (DIM), or a full agonist (PCB126). This was followed immediately by treatment with either vehicle (DMSO, 0.5%) or a TCDD or a TCDF standard curve. After plates were incubated at 33°C for 24 hr, EGFP fluorescence was read with a fluorometric plate reader (Synergy 2, BioTek Instruments, Winooski, VT). The excitation and emission wavelengths were 485 nm (20 nm bandwidth) and 530 nm (25 nm bandwidth). For each plate, we subtracted the fluorescence measured in wells of untreated cells from fluorescence in experimental wells. Because gain settings on the plate reader varied, we report only relative fluorescence values. The eight replicates of each combination within a plate were averaged in each experiment, and each experiment was repeated at least three times. Detailed information on the assay and concentrations used in the factorial experimental design are provided in the Supplemental Material (doi:10.1289/ehp.0901312).

We analyzed toxicity after the fluorescence measurement by assessing thiazolyl blue tetrazolium bromide labeling. Only combinations that had no significant toxicity (i.e., labeling ≥ 85% of that in vehicle-treated wells) were used in the model analyses (data not shown). See Supplemental Material (doi:10.1289/ehp.0901312) for additional details.

### Mathematical models

The GCA equation for a combination of two agonists *A* and *B*, with individual concentration–response curves *f_A_*([*A*]) and *f_B_*([*B*]), is


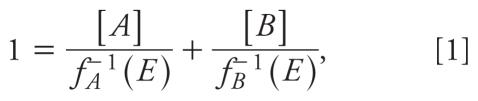


where *f_A_*^−^*^1^*(.) and *f_B_*^−^*^1^*(.) are the inverse functions of the individual concentration–response curves, and *E* is the effect level (Howard and Webster 2009).

**Figure 1:**
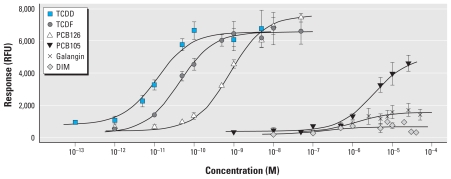
H1G1 concentration–response curves for experimental agents. Response is given in naïve- corrected relative fluorescence units. Lines are fits to Hill functions with a Hill parameter of 1.

We assumed that all concentration–response curves were Hill functions with Hill parameter 1: *f_A_*([*A*]) = α*_A_*[*A*]/(*K_A_* + [*A*]), where *K_A_* is the macroscopic dissociation equilibrium constant [equivalent to the effective concentration causing 50% of maximal response (EC_50_)], and α*_A_* is the maximal effect level of the ligand in the tissue or system under study. This function is usually a good fit for dioxin-like agents (Toyoshiba et al. 2004). Four-parameter Hill function fits of the individual concentration–response curves indicated that this was a reasonable assumption for our data (Figure 1). Some ligands showed a decline in reporter activity at the highest doses, a pattern also seen by Peters et al. (2006) and Nagy et al. (2002). This effect is not explained by frank toxicity or by the slight systematic variation in fluorometric readings across each plate. Because the decline may indicate a different mechanism of action, and as these points were inappropriate for fitting with a monotonic Hill function, we omitted them from Figure 1 and the analyses.

Substituting the inverse Hill function *f_i_*^−^*^1^*(*E*) = *E K_i_*/(α*_i_* *– E*) into Equation 1, we obtain the GCA model for combinations of *A* and *B:*


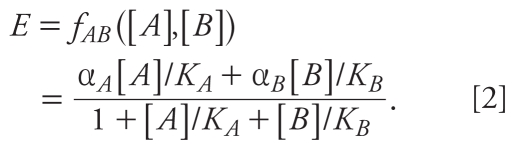


Isoboles of Equation 2 are found by solving for [*B*] and will always be straight lines. Under GCA, isoboles of agents with different maximal effects need not be parallel, but slopes can vary with effect level *E*:


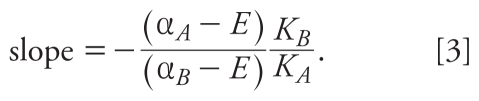


As shown by Equation 3, isoboles of this GCA combination are negatively sloped at low effect levels, flatten as the combination effect increases, and are positively sloped (like isoboles of a competitive antagonist) at effects above the maximal effect of the partial agonist α*_A_*. Under GCA, the relative potency (REP) of *A* compared with *B* at effect level *E* equals the negative of the slope for *E* ≤ α*_A_* and is largest in the limit of small effect: (α*_A_/*α*_B_*)(*K_B_/K_A_*).

The TEF method assumes that agent *A* has a concentration–response curve parallel to the reference agent *B* with the same maximum (α*_A_* = α*_B_*). The joint effect is given by


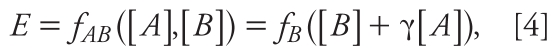


where γ is the TEF value, assumed constant for all effect levels: γ *= K_B_/K_A_*. For γ < 1, *A* acts as a diluted form of *B*. The TEF model is thus a special case of CA where all isoboles are parallel with slope –γ.

### Model fitting and significance testing

Individual (marginal) concentration–response functions were fit to a Hill function with Hill parameter *n=1*, using the optim() minimization function (specifying BFGS, a quasi-Newton method) in R (2.4.1; The R Project for Statistical Computing, http://www.r-project.org). The fluorescence value in the doubly unexposed well ([*A*]=0, [*B*]=0) was subtracted from the averaged combination matrices before fitting. The expected response surfaces for combinations were then calculated by substituting marginal fit parameters into Equations 2 and 4. For Equation 4, we used the experimentally determined REP value (i.e., the ratio of the EC_50_ values) for γ. Experimental and model response surfaces were plotted with the R wireframe() function and are linear interpolations between data points (at intersections of line segments). Isobolograms were generated with the R contour() function and are interpolated between data points.

To test the fit of the modeled response surface to the experimental data, we used the nonparametric Mann–Whitney test (Wilcoxon rank sum), performed using wilcox.test() in R. This statistic tests the hypothesis that the experimental data and modeled data come from the same distribution; a significant result (*p* < 0.05) indicates that the distributions differ. An alternative nonparametric test, Kolmogorov–Smirnov, produced similar results (data not shown). For visual inspection, the empirical cumulative distribution functions (ECDFs) of the experimental and model surfaces were plotted using plot(ecdf()) in R [see Supplemental Material, Figure 1 (doi:10.1289/ehp.0901312)].

The R code used in the analysis and plotting of the mixture data, along with a sample data set, is available on the Boston University Superfund Research Program web site (http://www.busrp.org/research.html).

## Results

### Characterization of single chemical dose–response curves

Treatment of H1G1.1c3 cells with TCDD, TCDF, PCB126, PCB105, or galangin each resulted in activation of the AhR, as indicated by the production of EGFP controlled by AhR transactivation. The data were well described by the assumed Hill functions (Figure 1). Table 2 shows the estimated maximal effect, EC_50_, and REP (the ratio of the EC_50_s) of each compound. As expected, TCDD, TCDF, and PCB126 were similarly efficacious at activating the AhR (Figure 1). The REP of PCB126 compared with TCDD, approximately 0.07, is near the 2006 World Health Organization (WHO) TEF value of 0.1 and is well within the order of magnitude accuracy expected for TEFs (Van den Berg et al. 2006).

The maximal effect of PCB105 was 61% of that elicited by TCDD, consistent with its characterization as a partial agonist. However, Peters et al. (2006) reported a maximal effect of approximately 11% of TCDD in this cell line after purification of commercial PCB105 with activated charcoal. The high potency of PCB126 compared with PCB105 (Peters et al. 2006) suggests that much of the activity of our sample is from the impurities rather than the target compound. Under GCA, the contaminated sample may be treated as a single agent, with potency and efficacy intermediate between that of pure PCB105 and pure PCB126. Support for this view is provided by the fact that our REP for PCB105, 5.4 × 10^−6^, is larger than the 1 × 10^−6^ reported by Peters et al. (2006) for purified PCB105. Both are less than the WHO TEF value of 3 × 10^−5^ for mono-*ortho* PCBs (Van den Berg et al. 2006).

The flavonoid galangin is found in two species of a gingerlike Asian root (*Alpina* spp.) (Ciolino and Yeh 1999). In the H1G1 assay, galangin significantly induced AhR reporter activity, although to only 30% of the level induced by TCDD (Table 2). Thus, galangin was characterized as a partial agonist in these experiments.

DIM is a selective AhR modulator found in cruciferous vegetables that has been studied as a possible chemopreventive agent for treating breast cancer (Hestermann and Brown 2003). DIM had little AhR-related activity in the H1G1 assay (approximately 8% of the maximal effect of TCDD), making it a nearly complete competitive antagonist (Table 2).

### TCDF + PCB126

**Figure 2:**
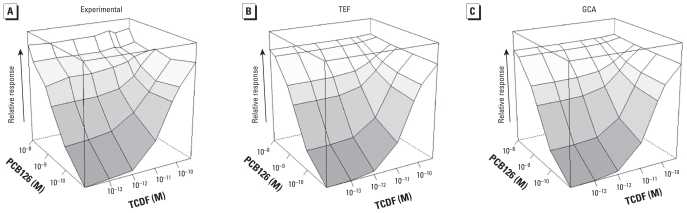
Response surfaces for TCDF plus PCB126 combinations shown for (*A*) experimental data, (*B*) TEF model prediction, and (*C*) GCA model prediction. TEF and GCA model surfaces were constructed from marginal concentration–response curves. The *x-* (TCDF) and *y-* (PCB126) axes are logarithmic, with zero dose plotted at 1/10 of the lowest dose; the *z-* (response, *E*) axis is linear. Concentrations were 10^−13^ to 10^−8^ M TCDF and 10^−10^ to 10^−8^ M PCB126.

We used mixtures of TCDF and PCB126 to assess the effects of a combination of two full agonists [see Supplemental Material, Table 1 (doi:10.1289/ehp.0901312) for means ± SEs]. Figure 2A shows the experimental response surface (the individual dose–response curves of TCDF and PCB126 are visible along the *x–z* and *y–z* faces, respectively). Figure 2B and C show expected results using the TEF and GCA approaches, respectively. Both models fit the data well (*p-*values for model rejection are 0.82 for TEF and 0.86 for GCA). For two full agonists, we expect the GCA model to reduce to the TEF model.

### TCDD plus PCB105

**Figure 3:**
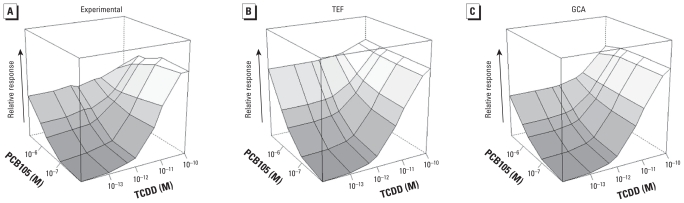
Response surfaces for TCDD plus PCB105 combinations shown for (*A*) experimental data, (*B*) TEF model prediction, and (*C*) GCA model prediction. TEF and GCA model surfaces were constructed from marginal concentration–response curves. The *x-* (TCDD) and *y-* (PCB105) axes are logarithmic, with zero dose plotted at 1/10 of lowest dose; *z-* (response, *E*) axis is linear. Concentrations were 10^−13^ to 2 × 10^−10^ M TCDD and 10^−7^ to 10^−5^ M PCB105.

For a combination of a full agonist and a partial agonist, we used TCDD and mono-*ortho*-substituted PCB105 [see Supplemental Material, Table 2, (doi:10.1289/ehp.0901312), for means ± SEs]. The experimental response surface is shown in Figure 3A, along with the TEF (Figure 3B) and GCA (Figure 3C) model fits. GCA fit the data better than the TEF model, although neither model was significantly rejected (*p-*values for model rejection are 0.08 for TEF and 0.63 for GCA). The PCB105 sample used for these experiments was contaminated with the more potent and efficacious PCB126. Because the PCB105 sample has relatively high efficacy, its competitive effect on TCDD is only moderate; consequently, the TEF model, although imperfect, is a reasonable description of the experimental data over a large range of effect.

### TCDD plus galangin

**Figure 4:**
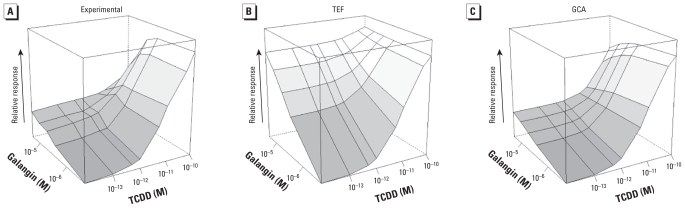
Response surfaces for TCDD plus galangin combinations shown for (*A*) experimental data, (*B*) TEF model prediction, and (*C*) GCA model prediction. TEF and GCA model surfaces were constructed from marginal concentration–response curves. The *x-* (TCDD) and *y-* (galangin) axes are logarithmic, with zero dose plotted at 1/10 of the lowest dose; *z-* (response; *E*) axis is linear. Concentrations were 10^−13^ to 2 × 10^−10^ M TCDD and 2.5 × 10^−6^ to 3 × 10^−5^ M galangin.

Galangin is a partial agonist of lower efficacy than PCB105 [see Supplemental Material, Table 3, (doi:10.1289/ehp.0901312), for means ± SEs]. The GCA model (Figure 4C) fit the experimental response surface (Figure 4A) extremely well (*p* for rejection = 0.79), whereas the TEF model was visibly inappropriate (Figure 4B) and was strongly rejected (*p* for rejection = 4 × 10^−5^). The difference between the models is apparent in Supplemental Material, Figure 1: The empirical cumulative distribution function (ECDF) for GCA closely follows the experimental data, whereas the TEF ECDF provides an extremely poor fit. The TEF model fails at high doses because it cannot take into account the reduced effect that occurs when the lower-efficacy galangin competes with TCDD for receptors.

**Figure 5:**
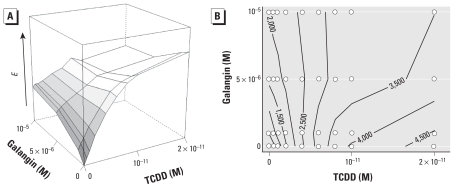
Response surface with arithmetic axes (*A*) and isobolographic analysis (*B*) of TCDD plus galangin combinations. For the isobologram of this surface (*B*), contours are interpolated between data points (circles). Arithmetic *x-* (TCDD) and *y-* (galangin) axes are identical in these plots. The GCA model predicts a vertical isobole near 5 × 10^−12^ M TCDD. Concentrations were 5 × 10^−13^ to 2 × 10^−11^ M TCDD and 10^−6^ to 10^−5^ M galangin.

Isoboles must be straight lines under GCA, but application of this visual test requires arithmetic *x-* and *y-*axes instead of the logarithmic axes commonly used in toxicology. We therefore performed additional experiments with a narrower range of linearly spaced doses (Figure 5A) [see Supplemental Material, Table 4, (doi:10.1289/ehp.0901312), for means ± SEs]. The isoboles of this surface (Figure 5B) approximately follow the GCA model, switching from negative to positive slopes near TCDD concentrations of 5 × 10^−12^ M as predicted by Equation 3. The isoboles are not perfectly linear because of minor variations in the response surface.

### TCDD plus DIM

**Figure 6:**
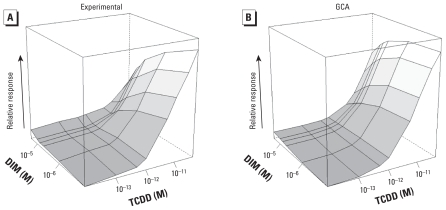
Response surfaces for TCDD plus DIM combinations shown for (*A*) experimental data and (*B*) GCA model prediction. The GCA model surface was constructed from marginal concentration–response curves. The *x-* (TCDD) and *y-* (DIM) axes are logarithmic, with zero dose plotted at 1/10 of lowest dose; *z-* (response, *E*) axis is linear. No TEF model was fit to these data. Concentrations were 10^−13^ to 10^−10^ M TCDD and 10^−6^ to 2 × 10^−5^ M DIM.

Figure 6A shows the experimental response surface for combinations of the full agonist TCDD and the nearly complete competitive antagonist DIM [see Supplemental Material, Table 5 (doi:10.1289/ehp.0901312) for means ± SEs]. As shown in Figure 6B, GCA fit the data well (*p* for rejection = 0.65). Because of the very low efficacy of DIM, we considered a TEF model inappropriate for these data.

## Discussion

Humans and wildlife are rarely exposed to individual PCBs, dioxins, or other AhR agonists. Most environmental exposures to these chemicals consist of at least dozens of individual agents with varying potencies and differing maximal effects. Risk assessment must describe the joint effects of such a mixture, whereas toxicologists need to assess the types of interaction occurring among chemicals in the mixture. The TEF approach estimates an equivalent combination dose using the concentrations and relative potencies of individual congeners, but it ignores differences in maximal effects, thereby failing to account for the competition of partial agonists for receptor sites. The presence of weak agonists is a known limitation of the TEF approach (Safe 1997); however, although mechanistic models analogous to that used here have occasionally been derived for other systems (e.g., effects of solvents on upper respiratory irritation in rats) under the name competitive agonism (Cassee et al. 1996), models that can account for the competitive effect of a partial agonist have only rarely been applied (Hestermann et al. 2000). Here, we show that GCA accurately predicts this effect.

We implemented the generic GCA model (Equation 1) for the case of two agents described by Hill functions with Hill parameter of 1 (Equation 2) and calculated the TEF model for the same agents (Equation 4). We tested the ability of each model to predict the effect on AhR activation of combinations of a full agonist with a full agonist, a full agonist with a partial agonist, and a full agonist with a nearly complete competitive antagonist. In each case, the GCA-modeled response surface fit the experimental data well. For lower-efficacy compounds, GCA fit the data substantially better than the TEF model. The TEF model was close to rejection for PCB105 and strongly rejected for galangin.

The isobologram, which is at the heart of the CA definition (Berenbaum 1989), was less useful in testing noninteractivity. Because the isobologram is plotted on the arithmetic scale of doses, it requires different dose selection within a much narrower range to evenly cover the appropriate dose–dose space. Calculation of isoboles requires interpolation of the response-surface contours from a relatively small number of doses (Figure 5); unfortunately, small variations in effect level cause variations in the response surface, which can distort isobole shapes. Instead, the response-surface plots, model fits, and nonparametric statistical tests appear to provide more robust visualization and analysis of the combination data.

### Utility of the GCA method

Use of the GCA method is not limited to the type of analysis described here. For instance, in some cases, GCA allows simple calculation of an equivalent dose of a reference agent. Suppose two agents *A* and *B*, described by Hill functions as above, occur in a mixture at constant dose ratio *c* = [*A*]/[*B*]. The mixture can be described as a single agent with summed dose [*X*]=[*A*] + [*B*] and parameters


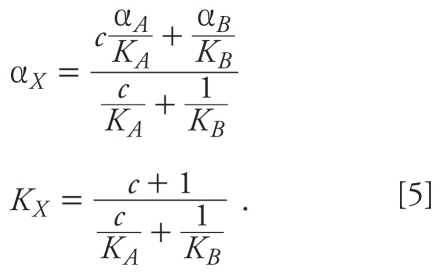


For example, a combination of equal doses of a full agonist *B* and a partial agonist *A*, where α*_A_* = 0.1, *K_A_* = *K_B_*, and *c*=1, yields a mixture (*X*) with a maximal effect α*_X_* = ^11^/_20_ α*_B_*.

Our specific implementation was for combinations of two agents described by Hill functions with Hill coefficient of 1, but the GCA model may, in principle, be used for any group of agents whose dose–response curves can be represented with appropriately invertible functions. Although we discussed only binary combinations, the GCA method can handle more complex mixtures. For combinations of agents *C_i_*, at effect level *E*, Equation 1 becomes


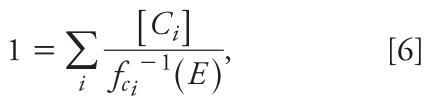


from which follow equations analogous to Equations 2 and 5 for treatment of multiple agents. In these ways, GCA lends itself to calculations of total effects of combination doses and to analysis of combinations containing more than two compounds. Thus, using straightforward modifications, the contexts in which GCA can be used are broadened.

### Implications for the TEF approach

The TEF approach assumes a constant REP at every effect level. By contrast, the GCA model predicts relative potencies that may vary with effect level. Because the TEF method does not account for the competitive effect of the partial agonist on the full agonist, it generally overestimates the joint effect compared with GCA. This difference is most pronounced at high effect levels and at higher relative concentrations of the partial agonist. For the concentration–response functions used here, a TEF model will always overpredict joint effects. For example, given a partial agonist *A* with a maximal effect 10% of full agonist *B* (α*_A_* = 0.1 α*_B_*), the TEF model overestimates joint effects by a factor of about 1.8 for combination doses given in the ratio [*A*]/[*B*] = *K_A_*/*K_B_* (i.e., each agent is dosed at a constant multiple of its EC_50_). For larger relative concentrations of partial agonist *A*, the TEF model overestimates the effect predicted by the GCA model by up to a factor of α*_B_/*α*_A_*; for larger relative concentrations of *B*, the partial agonist is less important and the TEF model approaches the GCA model.

For more complex mixtures, Equation 5 is easily extended, and the effective maximum becomes an average of the maxima of mixture components, weighted by the dose of each agent relative to its own EC_50_. As more partial agonists—or a higher dose of one partial agonist—are added to the mixture, the maximum effect of the mixture is reduced toward the efficacy of the least effective agent. For example, a mixture of one full agonist with ten 10% partial agonists (with each agent dosed at its own EC_50_) will have a maximal efficacy of 2/11 (18%). (The same result is obtained with one full agonist, dosed at its EC_50_, with one partial agonist, dosed at 10 times its EC_50_.) Although this particular result is within the order of magnitude accuracy attributed to the TEF approach (Van den Berg et al. 2006), the discrepancy between results of the GCA and TEF models would be even greater for mixtures that include numerous partial agonists or partial agonists in relatively high concentrations.

It has long been recognized that the TEF assumption of parallel dose–response curves may not be appropriate for some PCBs. Both individual PCB congeners and Aroclor mixtures have been shown to antagonize the AhR activity of full agonists such as TCDD (Aarts et al. 1995; Safe 1998); consequently, “the TEF approach overestimates the toxicity of these mixtures” (Harper et al. 1995). GCA would be expected to predict the lower effect levels of mixtures of low-efficacy PCBs and full AhR agonists. Further work will be required to test whether a GCA model, when applied to the specific experimental combinations, can explain these nonadditive interactions (Harper et al. 1995). Indeed, the use of the terms “nonadditive” or “antagonistic” demonstrates the difficulty of clear terminology in this field. Because the GCA null model describes the decreased joint effect of combinations that include partial agonists or competitive antagonists, those combinations may be thought of as additive in the sense of following the null model. In such cases, use of the term “noninteractive” in place of “additive” may be clearer.

Finally, commercially available “pure” PCB congeners are often contaminated with other congeners. Specific values obtained from laboratory assays using contaminated samples are likely to overestimate both the potency and the maximal effect of partial agonist ligands, in turn overestimating the REP and TEF values. The competitive–antagonistic effect of PCBs that are partial agonists is likely to be even more important with pure samples than with estimates derived from contaminated samples.

In sum, we found that the GCA model fit experimental data on mixtures of a diverse set of AhR ligands better than the TEF model. An important limitation of our results is that we used an *in vitro* reporter assay that does not take into account *in vivo* metabolism and pharmacokinetics, particularly for less-persistent AhR ligands, and the potential dependence of maximal effect levels on the choice of end point. Nevertheless, some of the PCBs currently classified as dioxin-like compounds and provided with TEFs appear to exhibit some degree of partial agonism (Aarts et al. 1995; Safe 1998). Our results suggest that application of TEFs to mixtures containing these compounds will tend to overestimate effects, with the discrepancy increasing with dose or relative amounts of partial agonists and antagonists. The implications depend on the use of the analysis. For judging toxicologic interaction (i.e., additivity vs. nonadditivity), we believe GCA will provide greater insight than the TEF model. For this purpose, it is essential to measure both REP and efficacy. For risk assessment of environmental exposures, use of TEFs may provide an adequate estimate at low doses, given the goal of an order of magnitude accuracy of the current system and the lack of an established database of efficacies similar to the existing database of potencies. For the dose–response curves we evaluated, the TEF system would provide a conservative (i.e., high) estimate of the toxicity of the mixture, a commonly accepted practice in risk assessment. In the long run, we believe that risk assessment of AhR ligands and other types of mixtures would benefit from increased attention to partial agonism. The GCA model provides one approach.

## Conclusions

GCA predicted joint effects of full agonist, partial agonist, and near-competitive antagonist combinations of AhR ligands. This approach has the potential to improve analysis and risk assessment of mixtures currently modeled with the TEF method. The GCA approach is not a mechanistic model but a broadly applicable definition of noninteraction. Consequently, GCA may be useful for analyzing other systems with different dose–response curves, possibly including estrogenic agents or phthalates. The GCA approach may be useful in classifying interactions of mixtures as well as in making predictions about their effects when the individual dose–response curves are well known.

## Figures and Tables

**Table 1:** Isobole shapes for special cases of GCA of two agents described by Hill functions (Hill parameter = 1).

Case	Slopes of isoboles^a^
CA	Negative, not necessarily equal
TEFs	Negative and equal
Competitive antagonism	Positive
Partial agonist	Negative below maximal effect level of partial agonist, positive above

a Under GCA, all isoboles are linear.

**Table 2:** Maximal effect and potency for individual compounds.

AhR ligand	Reference compound	Maximal effect (%)	EC_50_ (M)	REP^a^
TCDF	—	100	2.9 × 10^−11^	
PCB126	TCDF	99	4.1 × 10^−10^	7.1 × 10^−2^
TCDD	—	100	7.6 × 10^−12^	
PCB105	TCDD	61	1.4 × 10^−6^	5.4 × 10^−6^
TCDD	—	100	9.9 × 10^−12^	
Galangin	TCDD	30	4.1 × 10^−6^	2.4 × 10^−6^
TCDD	—	100	9.1 × 10^−12^	
DIM	TCDD	8^b^	6.6 × 10^−6^	1.4 × 10^−6^

Response characteristics for the tested ligand and the reference compound in each combination were determined by fitting dose–response data (see Figure 1) using a four-parameter Hill function.

a Experimental potency relative to reference compound (calculated as EC_50_ of reference divided by EC_50_ of compound) used for TEF models.

b Because initial curve fits extrapolated to an unreasonably high maximal response, the maximal response was set to the average value at the highest nontoxic dose and the EC_50_ value alone was fit.
